# Evaluation of cytogenetic damage in exfoliated nasal epithelial cells contributes to a better understanding of the pathogenesis of rhinosinusitis^☆^

**DOI:** 10.1016/j.bjorl.2019.11.004

**Published:** 2019-12-10

**Authors:** Ana Carolina Flygare Souza, Carla Máximo Prado, Daniel Araki Ribeiro

**Affiliations:** a Universidade Federal de São Paulo (Unifesp), Departamento de Ciências do Movimento Humano, Santos, SP, Brazil; b Universidade Federal de São Paulo (Unifesp), Departamento de Biociências, Santos, SP, Brazil

*Dear Editor,*

We read the manuscript of Drumond et al.^1^ recently accepted for publication in the Brazilian Journal of Otorhinolaryngology titled “Micronucleus count in nasal epithelial cells from patients with chronic rhinosinusitis and polyps” with much interest. In this article, the authors were able to detect high frequencies of micronucleus on nasal cells of patients with rhinosinusitis when compared to controls. In this regard, we were able to formulate some questions in order to contribute to the present work.

In the Results section, it was mentioned that “MN was not increased in patients with AERD (*p* =  0.310). There was also no association between nasal surgery in the previous 5 years and MN count (*p* =  0.251)”. From our point of view, it would be interesting to present these data in the manuscript, even if there is no statistical difference.

In this study, the experimental group was older than the control group. Significant statistically differences (*p* <  0.05) were found between groups. Taking into consideration that micronucleus frequency increases with increasing age, the authors justify such preferential treatment by means of three References in Discussion, but some of them were not properly interpreted. For example, the article published by Squier and Kremer (Reference number 24) did not investigate micronuclei in the exfoliated epithelial cells.^2^ Moreover, the paper published by Calderon-Garciduenas (Reference number 26) did not evaluate the micronucleus assay.^3^

Tolbert et al.^4^ have introduced some metanuclear changes indicative of cytotoxicity for the micronucleus assay in exfoliated cells, such as pyknosis, karyolysis, and karyorrhexis. The approach is important because cytotoxicity is a confounding factor for mutagenicity studies.^5^ For example, if cytotoxicity is increased the micronucleus frequency decreases because micronucleated cells are lost as a result of cellular death. Therefore, it would be interesting to know if and to what extent patients with rhinosinusitis present increased cytotoxicity as well as to evaluate the biological consequences of this on micronucleus incidence. Certainly, cytotoxicity is present in nasal mucosa from patients with chronic rhinosinusitis because this pathological condition induces cellular death as a result of inflammatory process.^6^

In any case, the results of this study clearly indicate that people suffering chronic rhinosinusitis have high a frequency of micronucleus in nasal cells. This means that genomic instability is present in these patients. It has been well established that genomic instability plays a crucial role in the pathogenesis of chronic degenerative diseases, such as cancer. For this reason, further studies investigating chromosome damage as well as cellular death from other contexts and paradigms in these patients are very important for better understanding the pathogenesis of rhinosinusitis.

## Funding

DAR and CMP are recipients of CNPq (Conselho Nacional de Desenolvimento Cientifico e Tecnologico) productivity fellowships (Financial code #001)

## Conflicts of interest

The authors declare no conflicts of interest.
